# Bias, the unfinished symphony

**DOI:** 10.11613/BM.2022.030402

**Published:** 2022-10-01

**Authors:** Abdurrahman Coşkun

**Affiliations:** 1 Acibadem Labmed Clinical Laboratories, Istanbul, Turkey; 2 Department of Medical Biochemistry, School of Medicine, Acibadem Mehmet Ali Aydinlar University, Istanbul, Turkey

**Keywords:** bias, pooled variances, sigma metric

## Abstract

In laboratory medicine, mathematical equations are frequently used to calculate various parameters including bias, imprecision, measurement uncertainty, sigma metric (SM), creatinine clearance, LDL-cholesterol concentration, *etc*. Mathematical equations have strict limitations and cannot be used in all situations and are not open to manipulations. Recently, a paper “Bias estimation for Sigma metric calculation: Arithmetic mean *versus* quadratic mean” was published in Biochemia Medica. In the paper, the author criticized the approach of taking the arithmetic mean of the multiple biases to obtain a single bias and proposed a quadratic method to estimate the overall bias using external quality assurance services (EQAS) data for SM calculation. This approach does not fit the purpose and it should be noted that using the correct equation in calculations is as important as using the correct reagent in the measurement of the analytes, therefore before using an equation, its suitability should be checked and confirmed.

## To the Editor,

**Figure 1:**
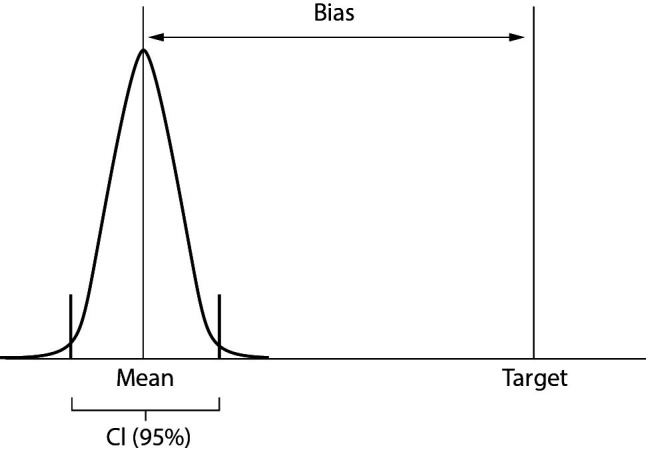
Bias is the mean of the replicate measurement results minus a reference quantity value. CI – confidence interval.

I read the paper “Bias estimation for Sigma metric calculation: Arithmetic mean *versus* quadratic mean” written by Ercan *et al.* with great interest (*1*). In the paper, the author criticized the approach of taking the arithmetic mean of the multiple biases to obtain a single bias and proposed a new method (quadratic mean) to estimate the overall bias using external quality assurance services (EQAS) data for sigma metric (SM) calculation (*2*). The bias issue has been a kind of unfinished symphony and is rarely evaluated correctly. As stated by Galileo Galilei “the book of nature is written in the language of mathematics”. Mathematic is the map of scientists but can cause chaos if not used properly. I agree with the author that taking the sum of biases may underestimate the overall bias, but the approach proposed by the authors of both papers contain major scientific errors as briefly summarized below:

First of all, bias cannot be calculated using a single measurement result. Unfortunately, in the literature, there are many papers containing this error and most of the authors use it without checking its scientific background. According to the International Vocabulary of Metrology (VIM) bias is the “average of the replicate indications minus a reference quantity value” (*3*). From this definition, we can say that two main components are essential to calculate the bias of an instrument correctly. The first one is the reference value and the second one is the average of replicate measurement results (Figure 1). The reference value should be obtained from certified reference materials and reference methods. However, if this is not possible then a consensus value can be accepted as the target value. In this case, the peer group’s average can be considered as the target value. In practice, laboratories usually use the peer group’s average as the target value, and this is correct. On the other hand, laboratories usually report single measurement results to evaluate the performance of the measurement procedure. Due to random error, using a single measurement result is not adequate to calculate bias and at least a duplicate measurement is necessary.

Additionally, the significance of the bias should be considered in all calculations related to bias (*4*). Since bias is “the difference between reference quantity and the average of repeated measurements of the measurand”, this difference might not be significant in all situations and therefore, before handling bias for further calculation its significance should be evaluated. If bias is not significant then it can be neglected.

The second important point is that there is no universal equation to be used in all different conditions. Therefore, before using an equation, its pros and cons should be considered. The equation used by the author is the simplified version of the equation of the pooled variances. The correct form of this equation (Eq.) is given below:



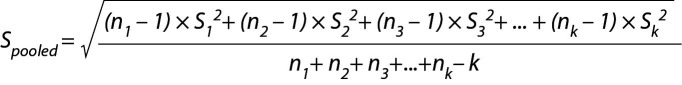



If the number of data used to calculate each variance is equal, *i.e.,* n_1_ = n_2_ = n_3_ = …= n_k_ then this equation can be simplified as given below:



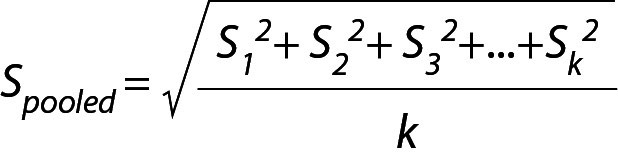



The components of equations 1 and 2 are variances. If the number of data (n) used to calculate each variance is different than n should be included and additionally, the variances must be random and homogenous. Otherwise using this equation may give incorrect results.

The third point is that even if the bias is calculated correctly, using bias as a linear component in SM calculation is not correct (*5*). The six sigma theory is based on the normal distribution and the curve of the normal distribution is bell-shaped. Bias can be treated as a linear component in uniform distribution but not in a normal distribution (*6*). Using bias as a linear component in SM calculation (see Eq. 3.) usually causes an underestimate of the performance calculation of the process. It is unfortunate that the performance of medical instruments calculated using this equation is very low. All these instruments are high-tech instruments, and their actual quality level is higher than the SM calculated using this equation.



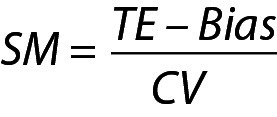



The fourth point is that if bias is known, it should be corrected. It does not make sense to include a known bias in the SM calculation while it can be corrected. In routine practice, we do not know whether bias is present or not if the process is not real-time monitoring. Therefore, in the six sigma methodology 1.5 standard deviation (SD) bias is included in the calculation. However, this 1.5 SD is not included directly as a linear parameter using the Eq. 3. In case of the presence of bias, the defects *per* million opportunities (DPMO) corresponding to SM are calculated using the mathematics of the normal distribution curve (*7*, *8*).

In conclusion, before using an equation, its suitability should be checked and confirmed. It should be noted that using the correct equation in calculations is as important as using the correct reagent in the measurement of the analytes.
